# Abstinence scoring algorithms for treatment of neonatal opioid withdrawal syndrome (NOWS)

**DOI:** 10.1038/s41372-024-01895-6

**Published:** 2024-02-16

**Authors:** Brenton A. Maisel, Susan C. Adeniyi-Jones, Eric Selvage, Samuel Ng, Walter K. Kraft, Inna Chervoneva

**Affiliations:** 1 Department of Neurology, University of California, Irvine, CA USA; 2 Sidney Kimmel Medical College, Thomas Jefferson University, Philadelphia, PA USA; 3 Nemours DuPont at Jefferson, Philadelphia, PA USA

**Keywords:** Paediatrics, Diseases, Medical research

## Abstract

**Objective:**

Chervoneva et al. (2020) developed an abbreviated score (sMNAS-9) derived from full modified Finnegan MOTHER NAS scale (MNAS) for evaluating severity of NOWS. We sought to develop NOWS treatment algorithms for clinical decision rules based on scores utilizing the shorter sMNAS.

**Study design:**

This was a retrospective study of 373 infants with NOWS scored with MNAS and treated with morphine between 2007 and 2016. The infants were randomly split into training/test sets. The training set was used to derive optimized cutoff values for sMNAS-9 scores. The independent set evaluated the sMNAS-9 clinical decision rules based on full MNAS in NOWS morphine and buprenorphine treatment algorithms.

**Result:**

Clinical decision rules based on sMNAS-9 yielded sensitivities of 88% or higher and specificities of 85% or higher for predicting the respective rules based on full MNAS.

**Conclusion:**

The sMNAS-9 scoring instrument is expected to yield similar clinical decisions in treatment of NOWS.

## Introduction

Neonatal opioid withdrawal syndrome (NOWS) follows in utero exposure to opioids. Signs of opioid withdrawal are manifest in autonomic, neurologic, and gastrointestinal domains with a recent expert panel consensus of at least 2 of 5 signs: excessive crying, fragmented sleep, tremors, increased muscle tone, and/or gastrointestinal dysfunction as well as a history of in utero exposure required for a diagnosis of NOWS [1].

A widely used scoring instrument to assess severity of neonatal opioid withdrawal is the Finnegan neonatal abstinence scoring system (FNAS) which includes 21 scored elements [2, 3]. One variant with a high degree of overlap is the MOTHER NAS scale (MNAS) with 19 scored elements [4]. Other short variants of FNAS with a goal of reducing the scoring burden while maintaining diagnostic accuracy have been developed. Maguire developed a seven-element FNAS score [5], Devlin generated an 8-element score [6], and Gomez-Pomar developed a 10-element score [7] with excellent specificity and negative predictive value for identifying infants with FNAS scores ≥8 and ≥12, which are common cutoffs used to guide dosing adjustments in neonates requiring pharmacotherapy. These shortened scores varied in the number of elements and the specific elements chosen. The gestational ages used were ≥36 (Devlin), ≥37 (Gomez-Pomar) and mixed in Maguire (though ~95% were ≥37 weeks).

We previously developed nine-element short scale scoring systems that maintained high sensitivity and specificity for matching the key dichotomized FNAS/MNAS scores of *≥* 8 and *≥* 12. While differences in study populations make direct comparisons difficult, these two nine-element versions, sFNAS-9 and sMNAS-9, had better test characteristics than the seven-element score proposed by Maguire, and were similar in performance to the 10-element score of Gomez-Pomar, except in improved sensitivity for predicting > 12 decision points in the full FNAS score. Furthermore, the 9-element shortened MNAS includes all 5 clinical signs determined characteristic of NOWS by consensus [1]. The MOTHER NAS scale (MNAS) and 9-element MNAS score (sMNAS-9) are shown in Table 1 (reproduced from [8]).

**Table 1:** The full MOTHER NAS instrument (MNAS) and shortened instrument (sMNAS-9).

Signs and Symptoms	Severity	Score	
		MNAS	sMNAS-9
Crying	Excessive high pitched Continuous high pitched	2 3	2 3
Sleeps	<1 h after feeding <2 h after feeding <3 h after feeding	3 2 1	3 2 1
Moro Reflex	Hyperactive Markedly Hyperactive	1 2	
Tremors: Disturbed	Hands or feet only, up to 3 s Arms or legs, over 3 s	1 2	
Tremors: Undisturbed	Hands or feet only, up to 3 s Arms or legs, over 3 s	1 2	1 2
Increased Muscle Tone	Difficult but possible to straighten arm and head lag present	1	1
	Unable to straighten arm and head lag absent	2	2
Fever >37.3 C (99.2 F)		1	1
Tachypnea	Respiratory Rate >60/mm	2	2
Poor feeding	Takes >20 min, uncoordinated, takes small volume, frequent stops to breathe	2	2
Vomiting (or regurgitation)	Vomits whole feeds, or at least x 2/feed when not burping	2	2
Loose Stools	Diaper is half liquid/half solid ± water ring	2	2
Excoriation	Skin is red but intact or healing, no longer broken	1	
	Skin not intact	2	
Generalized Seizure	Eyes staring, rapid involuntary eye movements, chewing, back arching, fist clenching, tonic-clonic movements	8	
Frequent Yawning	4 or more successive times	1	
Sweating	Wetness on forehead or upper lip	1	
Nasal Stuffiness	Any nasal noise	1	
Sneezing (4 or more successive times)	4 or more successive times	1	
Failure to thrive	Current weight ≥10% below birth weight	2	
Excessive Irritability	Consoling calms infant in 5 min or less	1	
	Consoling calms infant in 6–15 min	2	
	Consoling takes >15 min or is unsuccessful. Baby is sensitive or aversive to sound, light touch or, unable to calm by self.	3	
	Summed Score	0–43	0–19
Recorded but unscored elements			
Myoclonic jerks, Mottling, Convulsions, Fever 38.4 C (101.2 F), Retractions, Nasal flaring,			
Excessive Sucking, Projectile Vomiting, Watery Stools. All noted as present or absent			

(Reproduced from ref. [7])

**Fig. 1:**
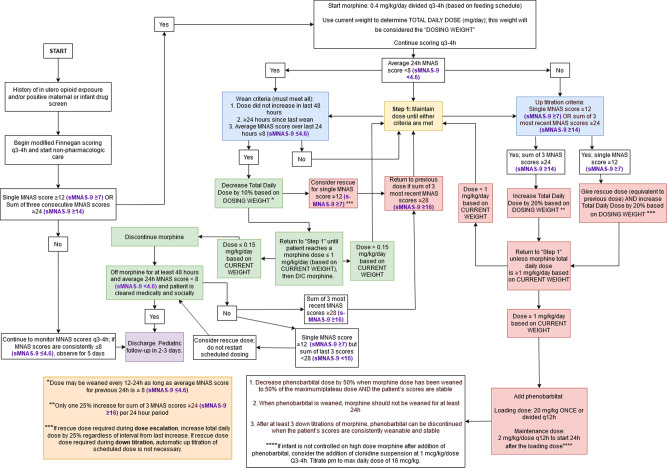
Algorithm for morphine treatment of NOWS based on full MNAS scoring and respective sMNAS-9 cutoff scores (purple, bolded).

**Fig. 2:**
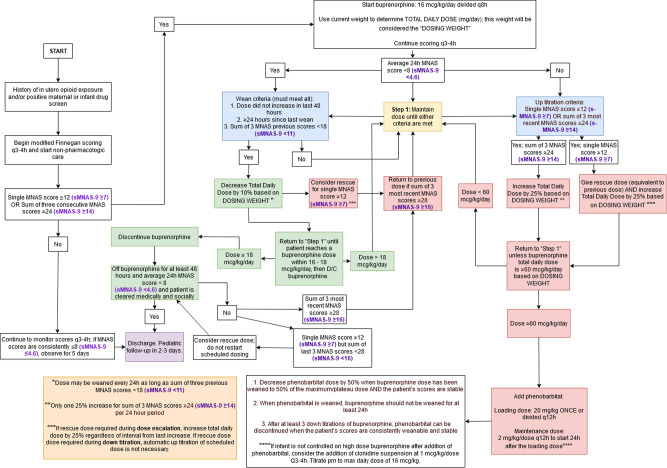
Algorithm for buprenorphine treatment of NOWS based on full MNAS scoring and respective sMNAS-9 cutoff scores (purple, bolded).

In the present work, we sought to develop new treatment algorithms with clinic decision rules based on cut off values for the short sMNAS-9 instrument while maintaining the integrity of the algorithms currently in use based on the original MNAS scoring. The Thomas Jefferson University Hospital Neonatal Intensive Care Unit (NICU) order set for morphine treatment of NOWS utilizing the original 19-item MNAS scoring tool was created in 2011 and refined into an algorithm in 2017 to reflect clinical practice in place since 2005 (Fig. 1) [4]. A similar algorithm for buprenorphine treatment was developed in 2018 after a successful randomized clinical trial established that treatment with sublingual buprenorphine resulted in a shorter duration of treatment and shorter length of hospital stay than treatment with oral morphine, with similar rates of adverse events [9]. The algorithm for buprenorphine treatment of NOWS based on full MNAS scoring is shown in Fig. 2. Our objective was to maintain the logic and clinical decision rules to increase, maintain, decrease the dose, begin or discontinue morphine or buprenorphine treatment while replacing the decision rules based on original MNAS scoring with decision rules based on cut-offs derived for sMNAS-9 scores. For this purpose, the thresholds based on longitudinal measures of full MNAS scores (single score, sums or averages of the last 3 scores or all scores within past 24 h) were considered “gold standard”, and similarly computed sums or averages of sMNAS-9 scores were used as predictors to optimize the cutoffs for sMNAS-9 that match as best as possible the cutoffs for full MNAS. The optimization of the cutoffs for sMNAS-9 was performed in the designated training set, and an independent test set was used to evaluate the agreement between the cutoffs for sMNAS-9 and the cutoffs for the full MNAS.

## Methods

This was a single center, retrospective study of infants with neonatal abstinence syndrome or NOWS treated at the Thomas Jefferson University Hospital between 2007 and 2016 with an ICD-9 code 779.5 (equivalent ICD-10 code p96.1). The study was approved by the Thomas Jefferson University Institutional Review Board with waiver of consent for a retrospective chart review. All infants requiring treatment for NOWS were admitted to the NICU. Treatment decisions based on the MOTHER NAS scale (MNAS) with 19 scored elements were summarized in existing local algorithms employing morphine and buprenorphine had been developed through local quality improvement initiatives. Phenobarbital was added as adjunct therapy for cases in which withdrawal symptoms remained severe despite maximal dose of primary opioid therapy. Algorithms were followed until infants weaned off morphine or buprenorphine (Figs. 1, 2). Nineteen scored elements of MNAS were evaluated every 3–4 h by trained nurses. Reliability of nurse scoring was ensured by periodic training sessions and nurse champions. The short sMNAS-9 scores were computed based on scores from a subset of items derived from MNAS tool as shown in Table 1. In this analysis, an average MNAS score ≥8 was equivalent to an sMNAS-9 score of ≥5 while an MNAS score of ≥12 corresponded to an sMNAS-9 of ≥7 rounded to the nearest integer [8]. The algorithm for computing longitudinal metrics for the decision rules based on full MNAS scoring and short sMNAS-9 scores was implemented in Python 3.7 with the pandas data frame package [10, 11]. Each infant was assigned an anonymous numerical ID tag in addition to the calculated modified Finnegan scores at each time point. The datasets were then filtered so that each infant’s scores were listed in chronological order. A timer was created which counted the time passing from the last dosage increase/decrease based on the algorithm’s rules where the initial score was determined to be at a time of 0. A column was generated which determined at each time point whether or not the dose increased, decreased, or remained the same. Based on these results, additional columns were generated using an indicator variable to determine at what timepoints the patient had already received a first dosage decrease, whether 48 h had passed since the last dosage increase, and whether 24 h had passed since the last dosage decrease.

A training set was created by randomly assigning infants to the training set with a probability of 0.5. The rest of the infants were assigned to the test set. For each infant, the longitudinal time- stamped measures of MNAS and sMNAS-9 during the treatment period were used to compute the average score within 24-h and the sum of the three past consecutive scores for each time when MNAS was evaluated. In the training set, classification and regression tree (CART) model [12] was used to predict cutoffs for MNAS used in the clinical decision algorithms (Figs. 1, 2). That is, the response variables for CART models were defined as indicators of (i) average 24 h MNAS score <8; (ii) average 24 h MNAS score ≤8; (iii) sum of 3 past consecutive MNAS scores <18; (iv) sum of 3 past consecutive MNAS scores ≥24; (v) sum of 3 past consecutive MNAS scores ≥28 (vi) single score of 12 or greater (a frequent criterion to initiate morphine or buprenorphine treatment or give rescue doses and needs to be included in the model). Separate CART models were used for each indicator with the corresponding longitudinal sMNAS-9 metrics (score of ≥12, average 24 h, or sum of 3 past consecutive) as predictors.

In the training set, the optimal cutoffs for longitudinal sMNAS-9 metrics scales were selected to maximize the Youden index of the empirical ROC curves for predicting each indicator based on the MNAS score. The Youden index is the sum of the sensitivity and specificity of the dichotomized predictor. Then the performance of the optimal cutoffs for longitudinal sMNAS-9 metrics was evaluated in the test set. The sensitivity and specificity were estimated with the corresponding bootstrap-based 95% confidence intervals using the R package ‘pROC’ [13]. Statistical analyses were performed in R [14]. corresponding to the highest Youden index.

## Results

The training set included 183 infants with 31,772 MNAS scores, and the test set included 190 infants with 31,710 MNAS scores. Table 2 shows the optimal cutoffs for the longitudinal sMNAS-9 metrics for each of the clinical decision rule based on full MFNAS scale derived using the training data. The cutoffs for the average 24-hour scores were rounded to the nearest tenth. Table 2 shows the sensitivity and specificity with corresponding confidence intervals corresponding to using the longitudinal sMNAS-9 metrics with the optimal cutoffs for predicting clinical decisions based on the full MNAS scores computed in the independent test set. In the test set, all estimated specificities were 85% or higher, and all estimated sensitivities were 88% or higher and all but one were significantly higher than 90% (the lower limit of the 95% CI above 90%). Adopting the new optimal cutoffs for longitudinal sMNAS-9 metrics, the algorithms for morphine and buprenorphine treatment are shown in Figs. 1, 2, respectively.

**Table 2:** Performance of clinical decision rules based on longitudinal sMNAS-9 metrics in the independent test set including 190 infants with 31,710 MNAS scores.

Full MFNAS criteria	Matching sMNAS-9 criteria	Sensitivity (95% CI)	Specificity (95% CI)
Average 24 h score <8	Average 24 h sMNAS-9 scores < 4.6	92.5% (92.2–92.8)	92.1% (91.2–93.4)
Single MNAS score ≥12 (*)	Single sMNAS-9 score ≥7	97.2% (96.4–98.0)	94.8% (94.7–95.0)
Average 24 h NAS score ≤8	Average 24 h sMNAS-9 scores ≤ 4.6	91.9% (91.6–92.2)	94.4% (93.5–95.3)
Sum of 3 consecutive MFNAS ≥ 24	Sum of 3 consecutive sMNAS-9 ≥ 14	92.9% (92.1–93.8)	89.7% (89.3–90.0)
Sum of 3 consecutive MFNAS ≥ 28	Sum of 3 consecutive sMNAS-9 ≥ 16	95.3% (94.1–96.4)	93.0% (92.7–93.3)
Sum of 3 consecutive MFNAS < 18	Sum of 3 consecutive sMNAS-9 ≤ 10	88.6% (88.2–89.1)	85.4% (84.7–86.0)

(*) Performance of clinical decision rules based on single sMNAS-9 score was previously reported in ref. [8].

## Discussion

In this work, we present the morphine and buprenorphine treatment algorithms for infants with NOWS based on a 19-item MNAS scoring tool [4] and 9-element MNAS score (sMNAS-9) [8]. Both algorithms follow the same logic, but the cut points for sums and averages are different for original MNAS and sMNAS-9 scales. The resulting decision rules based on sMNAS-9 scores demonstrate high sensitivity and specificity predicting the corresponding decision rules based on the original MNAS scale. Implementation of the proposed shorter scoring systems should maintain a reasonable degree of NOWS instrument performance while decreasing the effort needed from the care team and cost of monitoring prenatally opioid exposed infants. The strengths of our study include large data sets and high-quality measures collected at a single site eliminating implications of heterogeneity between sites.

Recent work by Young et al. of the Eat, Sleep, Console [15] demonstrated decreased overall length of stay of infants with NOWS mostly driven by reduction in the number of infants treated, an implicit result of this new approach. This ESC study did not show significant differences in length of stay for infants ultimately pharmacologically treated. Assuming the same density of non-pharmacologic care, it assumes that a reduction in the number of infants treated (allowing for higher symptom scores in untreated infants) will ultimately result in better overall outcomes. A follow up from this prospective, randomized trial is currently underway. A separate study reported significantly greater postnatal weight loss among infants managed with the ESC tool [16]. Our study cohort included only infants that were treated based on severity of NOWS signs assessed by MNAS while rooming in with their mothers before treatment started. It should be noted that our work derives from a single center and from a time not necessarily representative of current drug exposure patterns nor of the density of nonpharmacologic methods currently widely used. The ESC study of Young et al. was more recent, prospective and multicenter. In our work, we do not address the appropriateness of the NOWS severity criteria for starting treatments. Even if the cut point for initiation may ultimately be changed to align with ESC, the results of the ESC study suggest that cut points do not need to be adjusted which lends support to our current work. Unlike the ESC tool, the sMNAS-9 incorporates all five clinical signs deemed characteristic of neonatal withdrawal by expert consensus and is unlikely to undertreat infants. Furthermore, our prior work on developing a short MNAS scale appears to be largely consistent with results of other groups who have published on this topic.

## Data Availability

Available from the corresponding author upon request.
